# Scattering of Ultra-relativistic Electrons in the Van Allen Radiation Belts Accounting for Hot Plasma Effects

**DOI:** 10.1038/s41598-017-17739-7

**Published:** 2017-12-18

**Authors:** Xing Cao, Yuri Y. Shprits, Binbin Ni, Irina S. Zhelavskaya

**Affiliations:** 1 Department of Space Physics, School of Electronic Information, Wuhan University, Wuhan, China; 2 Helmholtz Centre Potsdam, GFZ German Research Centre for Geosciences, Potsdam, Germany; 3 Institute of Physics and Astronomy, University of Potsdam, Potsdam, Germany; 4 Department of Earth, Planetary, and Space Sciences, University of California, Los Angeles, California USA

## Abstract

Electron flux in the Earth’s outer radiation belt is highly variable due to a delicate balance between competing acceleration and loss processes. It has been long recognized that Electromagnetic Ion Cyclotron (EMIC) waves may play a crucial role in the loss of radiation belt electrons. Previous theoretical studies proposed that EMIC waves may account for the loss of the relativistic electron population. However, recent observations showed that while EMIC waves are responsible for the significant loss of ultra-relativistic electrons, the relativistic electron population is almost unaffected. In this study, we provide a theoretical explanation for this discrepancy between previous theoretical studies and recent observations. We demonstrate that EMIC waves mainly contribute to the loss of ultra-relativistic electrons. This study significantly improves the current understanding of the electron dynamics in the Earth’s radiation belt and also can help us understand the radiation environments of the exoplanets and outer planets.

## Introduction

Van Allen radiation belts were the first discovery of the space age. Quantifying the radiation belt dynamics has been a long-standing challenge due to competing acceleration and loss mechanisms. While radiation belt electron acceleration mechanisms have received much attention in recent years^1–3^, electron loss mechanisms remain to be fully understood. Two major mechanisms have been recognized to account for the loss of radiation belt electrons, including the pitch-angle scattering of electrons into the atmosphere by the resonant interactions with plasma waves and the losses to the magnetopause followed by outward radial diffusion^4–7^. While the losses to the magnetopause occur at a wide range of electron energy, wave-driven scattering of electrons can only contribute to the losses of electrons with energies higher than the minimum resonant energy (MRE). As a frequently observed wave mode in the Earth’s magnetosphere^8–10^, Electromagnetic Ion Cyclotron (EMIC) waves have long been recognized to efficiently scatter radiation belt electrons into the atmosphere via cyclotron resonant interactions^11^. Previous theoretical studies^11–14^ stated that EMIC waves should be responsible for the rapid scattering loss of MeV electrons in the outer radiation belt.

However, significant differences have recently been found between the dynamics of the relativistic and ultra-relativistic electron population. Using a comparison of global 3-D simulations and observed electron fluxes at MeV and multi-MeV energies, a recent study^15^ suggested that pitch-angle scattering driven by EMIC waves could provide an efficient mechanism for the loss of ultra-relativistic electrons, while the effects of EMIC waves are negligible for electrons below ~2 MeV. Both observations and modeling showed that EMIC waves could cause significant loss in the ultra-relativistic electron population over a broad range of pitch angles^16–18^. By comparing global observations with numerical simulations of radiation belt electrons for the January 17, 2013 storm, it was suggested that while the scattering loss due to EMIC waves contributes to the profound dropout of >4 MeV electrons, relativistic electrons below 2 MeV are unaffected and show an increase in fluxes^19^. A subsequent study^20^ clearly proved the role of EMIC waves in the rapid local loss of multi-MeV electrons by analyzing the radial profile of electron phase space density (PSD). They demonstrated that for the January 17, 2013 storm, electron PSD variations showed clear deepening minimums at high energies that can only be produced by EMIC wave-induced scattering.

In order to understand this discrepancy between previous theoretical studies and recent observations, we incorporate the hot plasma effects in evaluations of electron minimum cyclotron resonant energy, which denotes the minimum energy of electrons that can undergo cyclotron resonance with EMIC waves for scattering into the loss cone. We demonstrate that for all reasonable combinations of input parameters, EMIC waves mainly contribute to the loss of ultra-relativistic electrons, while the relativistic electron population is practically unaffected. Our theoretical results are in good agreement with recent observational studies.

## Results

**Figure 1:**
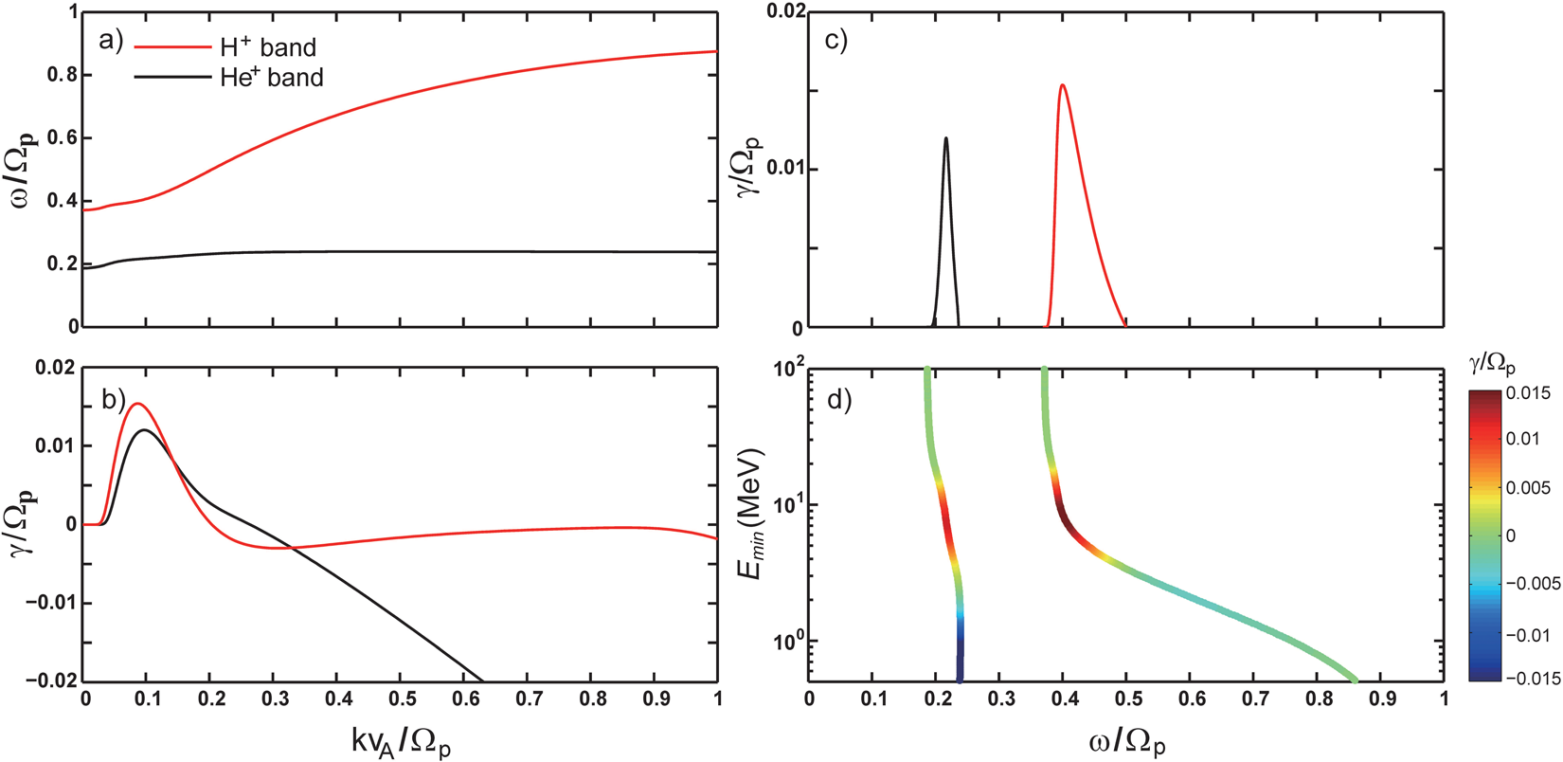
Calculation of electron MRE for interactions with EMIC waves. (**a**) Real frequencies and (**b**) linear growth rates as a function of wave normal. (**c**) Linear growth rates and (**d**) resonant energies as a function of wave real frequency. Wave real frequencies *ω* are normalized to the proton gyro-frequency Ω_*p*_ and wave number *k* is normalized to ${{\rm{\Omega }}}_{p}/{v}_{A}$, where Ω_*p*_ is the proton gyro-frequency and *ν*_*A*_ is the Alfvén velocity. The color bar in Fig. 1d denotes the corresponding growth rates. Red and black lines denote the results for H^+^ and He^+^ band EMIC waves, respectively. In this figure, L-shell is set as 4.5, the ambient magnetic field is assumed to be dipolar and electron density is adopted from an empirical density model^19^. We choose a typical set of ion composition ratios, in which O^+^/H^+^ and O^+^/H^+^ are respectively assumed to be 7.5% and 30%. We assume that only 10% of protons are hot with the parallel temperature *T*_*hp*_ of 25 keV and temperature anisotropy *A*_*hp*_ of 1.

By solving the linear dispersion relation of EMIC waves, we can obtain the real frequencies *ω*_*r*_ and temporal growth rate *γ*, as shown in the left panels of Fig. 1, in which the red lines denote the H^+^ band branch and the black lines denote the He^+^ branch. Note that O^+^ band EMIC waves are not taken into account in this study as they have significantly lower occurrence rates and weaker wave amplitude than the other two bands^21^. Following previous studies, we use a particular set of realistic parameters (see Methods). Although Fig. 1d shows that the resonant energy can be much lower than 1 MeV as the wave frequency approaches the ion gyrofrequencies, the corresponding growth rates are negative, indicating that waves cannot be excited at these wave frequencies. Therefore, following the previous study^22^, which was done before wave measurements became available, we obtain MRE for each wave band at the limit of marginal stability where the wave growth rate *γ* = 0. For the results presented in Fig. 1, the MRE is ~3.4 MeV for H^+^ and ~2.5 MeV for He^+^ bands, respectively.

**Figure 2:**
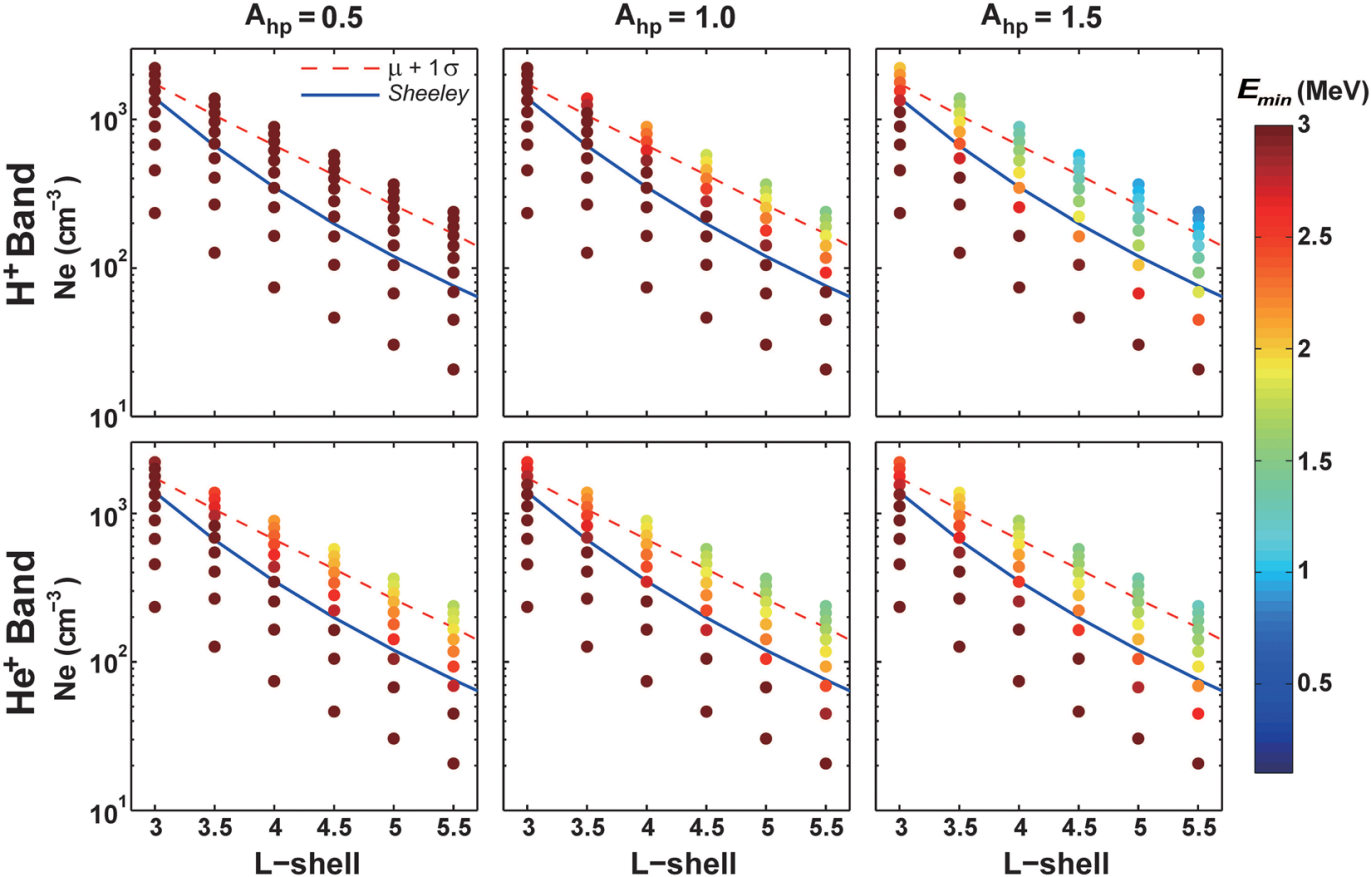
Sensitivity of electron MRE to hot H^+^ proton anisotropy. Electron MRE as a function of L-shell and electron density for different hot H^+^ temperature anisotropies (*A*_*hp*_ = 0.5, 1.0 and 1.5) corresponding to H^+^ and He^+^ band EMIC waves. L-shell is the radial distance in Earth radius (1 *R*_*E*_ = 6, 371 km) from the Earth’s center to the background field line. Red dashed lines denote the electron densities at one standard deviation above the mean value. Blue solid lines denote electron densities from an empirical density model^19^, which is frequently used as the statistical average value for density.

Figure 2 shows the scatter plot of MRE as a function of L-shell and electron number density corresponding to H^+^ and He^+^ band EMIC waves for three specific hot H^+^ anisotropies (*A*_*hp*_ = 0.5, 1.0 and 1.5). All other parameters (ion concentration ratio, hot H^+^ parallel temperature *T*_*hp*_ and hot H^+^ abundance *η*_*hp*_) are identical to those used to obtain Fig. 1. Due to the strong dependence of MRE on the ambient electron density^23^, we use electron densities over a broad spatial range inside the plasmasphere^24^. The red dashed lines correspond to plasmaspheric electron densities with one standard deviation above the mean electron density, which can be used as an upper limit estimate for density, and the blue lines correspond to plasmaspheric electron densities from an empirical plasmaspheric density model (hereinafter *Sheeley* model)^25^, which is frequently used to obtain the statistically average plasma density values inside the plasmasphere. Since the convection electric field that transports the source ions for the excitations of EMIC waves could also cause the convection and erosion of plasmaspheric electrons, the high density with one standard deviation above the mean value does not likely correspond to the presence of enhanced EMIC waves. Figure 2 shows that, for most typical and realistic values of hot H^+^ anisotropy and electron density, the electron resonant energy exceeds 2 MeV. Only when the anisotropy is very large and the electron density is unusually high (above one standard deviation), electron resonant energies for interactions with H^+^ band EMIC waves can fall below 1.5 MeV. Sensitivity simulations shown in Supplementary Figures 1 and 2 indicate that electron minimum resonant energy is insensitive to the assumed hot proton abundance or temperature.

**Figure 3:**
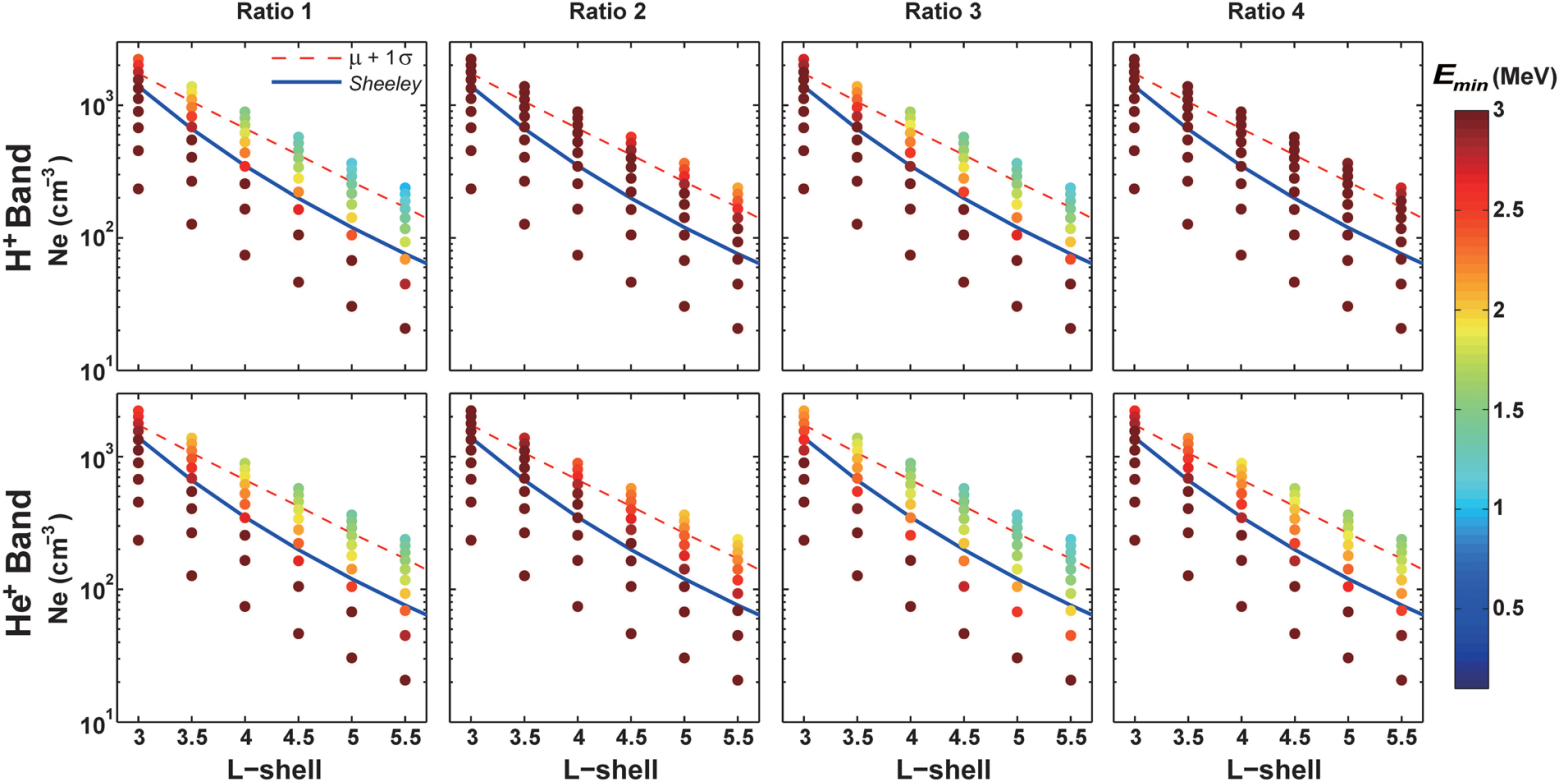
Sensitivity of electron MRE to ion concentration ratios. Electron MRE as a function of radial distance (L-shell) and electron density for different concentration ratios of H^+^, He^+^ and O^+^ ions (shown in Table 1) corresponding to H^+^ and He^+^ band EMIC waves. Red dashed lines denote the electron densities at one standard deviation above the mean value. Blue solid lines denote electron densities from an empirical density model^19^, which is frequently used as the statistical average value for density.

Figure 3 presents the sensitivity of electron MRE to the variation of ion concentration ratio. Four sets of ion concentration ratio are summarized in Table 1, which tabulates various combinations of typical ion concentrations^26^. All other parameters (parallel temperature *T*_*hp*_, temperature anisotropy *A*_*hp*_ and abundance *η*_*hp*_ of hot protons) are identical to those used to obtain Fig. 1. In order to obtain MRE much less than 2 MeV, O^+^ ion abundance should be very small and electron density should be much higher than typical values shown as blue lines.

**Table 1:** Four specific concentration ratios of H^+^, He and O^+^ ions adopted in the calculations of electron MRE due to EMIC waves.

*Ratio*	He^+^/H^+^	O^+^/H^+^
**1**	5%	10%
**2**	5%	50%
**3**	10%	10%
**4**	10%	50%

A summary of MRE corresponding to different H^+^ temperature anisotropies and different ion composition ratios in the heart of the outer radiation belt at L = 4.5 is shown in Table 2. The green values correspond to MRE obtained using electron densities with one standard deviation above the mean values, and the blue values correspond to MRE obtained using electron densities from the *Sheeley* model^25^. ‘Nan’ denotes that MRE is larger than 10 MeV, or EMIC waves cannot be excited. Although we show that EMIC waves can cause scattering losses of <2 MeV electrons, such scattering of low-energy electrons requires certain extreme conditions, such as large temperature anisotropy, high electron density and low O^+^ ion abundance. Results of MRE for electron density models with one standard deviation and of the *Sheeley* model are obtained by setting the perpendicular velocity of electrons ${v}_{\perp }=0$, which indicates that the corresponding equatorial pitch angle *α*_*eq*_ = 0°. The pitch-angle scattering by EMIC waves cannot take place at all *α*_*eq*_ for electrons with energy lower than MRE. However, EMIC waves should be able to scatter electrons with relatively large *α*_*eq*_ in order to cause significant losses of the major population of radiation belt electrons due to the fact that electrons with low *α*_*eq*_ only contribute a small fraction to the total electron population. In Table 2, we also show MRE for electrons with *α*_*eq*_ = 30°. It is indicated that MRE for *α*_*eq*_ = 30° is above ~2 MeV, suggesting that EMIC waves mainly contribute to the significant loss of the ultra-relativistic electron population.

**Table 2:**
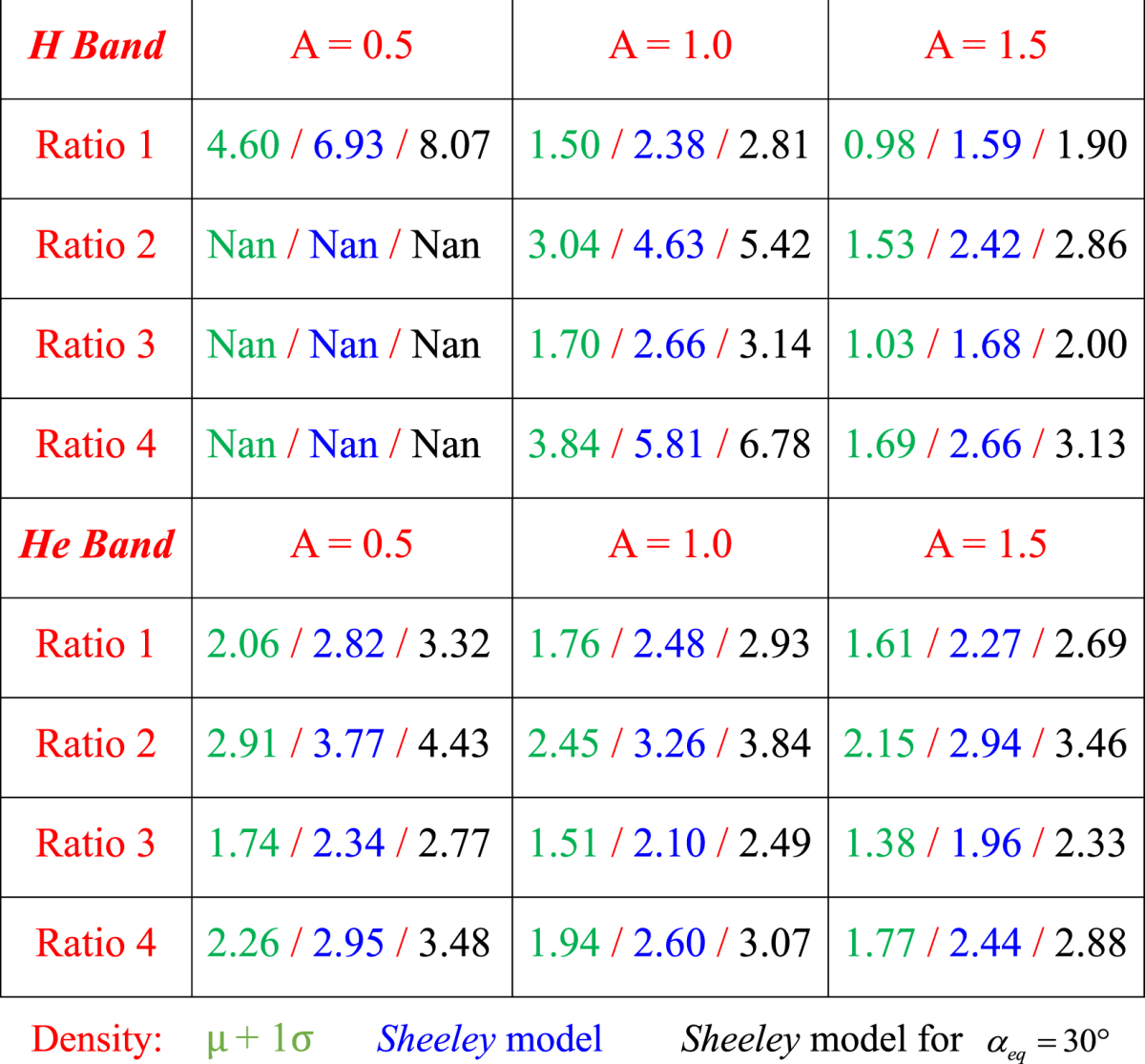
Electron MRE corresponding to different hot H^+^ temperature anisotropies and ion concentration ratios for (top) H^+^ band and (bottom) He^+^ band EMIC waves at the heart of outer radiation belt (L-shell = 4.5). Green values indicate MRE corresponding to electron density at one standard deviation above the mean value. Blue values indicate MRE corresponding to electron density from the Sheeley model. Black values indicate MRE of electrons with equatorial pitch angle *α*_*eq*_ = 30°.

## Discussion

This study shows that electron MRE for cyclotron resonant interactions with EMIC waves are typically larger than 2 MeV. The presented results correspond to representative, average ambient plasma (plus one σ uncertainty) and dipolar geomagnetic field. During some geomagnetic storms and substorms, plasma conditions may be very different from statistical averages. It is worthwhile to note that propagation of waves may also affect resonance conditions and very high plasma density in plasmaspheric plumes and unusually high anisotropies may potentially result in lower MRE below 2 MeV.

Since electrons with energies higher than MRE can be scattered more efficiently over a broader pitch-angle range^12^, EMIC waves can result in much more significant loss of higher energy electrons. Accurate estimation of loss and the minimum energy at which EMIC induced loss will be significant requires quantifications of wave induced electron diffusion coefficients and inclusion of these coefficients into 3-D diffusion codes, which should be the subject of future studies.

Previous observations showed that the Earth’s radiation belts are devoid of >10 MeV electrons. Scattering of electrons into the atmosphere by EMIC waves could be an important limiting factor for the upper energy limit of the trapped electron population. Ultra-relativistic electrons >10 MeV are observed in the radiation belts of outer planets, such as Jupiter and Saturn^27,28^. We suggest that this may be due to the absence of EMIC waves in these planets, or much higher electron MRE that is larger than 10 MeV. Higher MRE in the magnetospheres of Jupiter and Saturn may be due to a stronger background magnetic field intensity, which can raise MRE. Additionally, it should be noted that the devoid of >10 MeV electrons in the Earth’s outer radiation belt may also be due to lack of an efficient mechanism to energize electrons up to very high energies (i.e., >~10 MeV).

This study is relevant for the broader astrophysics community, as it gives quantitative estimates of electron energies that can be scattered by EMIC waves. Hence, efficient scattering by EMIC waves may impose the upper limit on radiation belt electron energies and also inhibit electron acceleration processes in the magnetospheres of the planets and exoplanets.

## Methods

Assuming that particle populations can be described by bi-Maxwellian distributions^29–31^, the full kinetic linear dispersion relation of left-hand parallel propagating EMIC waves can be written as follows^31–33^,

1 $$0=D(\omega ,k)={\omega }^{2}-{k}^{2}{c}^{2}+\sum _{s}{\omega }_{ps}^{2}({A}_{s}+(({A}_{s}+1)(\omega -{{\rm{\Omega }}}_{s})+{{\rm{\Omega }}}_{s})\frac{Z({\zeta }_{s})}{k{\alpha }_{||s}}),$$

where the wave frequency $\omega ={\omega }_{r}+i\gamma$ is complex, consisting of the real part *ω*_*r*_ and imaginary part *γ*, *k* is the wave number, *c* is the speed of light, ${{\rm{\Omega }}}_{s}={q}_{s}B/{m}_{s}$ is the signed gyrofrequency and ${\omega }_{ps}={({n}_{s}{q}_{s}/{m}_{s}{\varepsilon }_{0})}^{1/2}$ is the plasma frequency of multiple species (e^−^, H^+^, He ^+^ and O^+^), temperature anisotropy ${A}_{s}={T}_{\perp s}/{T}_{||s}-1$, thermal velocity ${\alpha }_{||s}={(2{T}_{||s}/{m}_{s})}^{1/2}$, ${T}_{\perp s}$ and ${T}_{||s}$ are the perpendicular and parallel temperature, and ${\zeta }_{s}=(\omega -{{\rm{\Omega }}}_{s})/({k}_{||}{\alpha }_{||s})$ is the argument of plasma dispersion function *Z* ^34^.

By solving the linear dispersion relation, a previous study^22^ investigated electron resonant energies at the limit of marginal stability and suggested that EMIC waves can only resonate with highly relativistic electrons. However, heavy ions such as He^+^ and O^+^ ions, which profoundly influence the generation and propagation of multi-band EMIC waves, were not taken into account in the calculations, as those measurements were not available at that time. Specifically, the abundance of O^+^ ions has been found to be highly variable in response to geomagnetic activities^26,35^, indicating the vital importance of including O^+^ ions in the resonant interactions between radiation belt electrons and EMIC waves.

In Fig. 1, we start by performing calculations for a particular set of realistic parameters. L-shell is set as 4.5, representative of the heart of the outer radiation belt. The background magnetic field is assumed to be dipolar, and electron density *N*_*e*_ is adopted from an empirical plasmaspheric density model^19^. We choose a typical set of ion composition ratios, in which O^+^/H^+^ and O^+^/H^+^ are respectively assumed to be 7.5% and 30%, following the statistical study of the ion composition over a solar cycle in the inner magnetosphere^20^. In addition to four cold particle species (cold e^−^, H^+^, He^+^ and O^+^), hot anisotropic ring current protons are also included to provide the free energy for the generation of EMIC waves. Following previous studies^31,33,36–39^, we assume that all cold particle species are isotropic with the temperature of 1 eV and that only 10% of protons are hot with the parallel temperature *T*_*hp*_ of 25 keV and temperature anisotropy *A*_*hp*_ of 1. When *k* and *ω*_*r*_ are available, by setting the electron perpendicular velocity ${v}_{\perp }=0$, we can easily obtain the electron MRE for EMIC waves^23^.

The upper and lower boundaries of electron densities in Figs 2 and 3 are adopted from a recent density model^24^, which were based on an algorithm that automatically determines the upper hybrid frequency and infers the electron densities from the Electric and Magnetic Field Instrument Suite and Integrated Science (EMFISIS) electric field measurements on the Van Allen Probes. While the lower boundary corresponds to lowest value of electron density from this density model, the upper boundary corresponds to electron density with two standard deviations above the mean value. The red dashed lines denote electron density with one standard deviation above the mean value, which could be used as an upper limit estimate for density. It should be noted that the blue lines are adopted from the *Sheeley* model^25^, which is frequently used as the statistically average electron density.

### Data availability

The electron density used in this paper is from NURD data set, which is available from ftp://rbm.epss.ucla.edu/ftpdisk1/NURD.

## Electronic supplementary material

Supporting Information
