# Supplementary material for: Scattering of Ultra-relativistic Electrons in the Van Allen Radiation Belts Accounting for Hot Plasma Effects
Source: Sci Rep. 2017 Dec 18;7:17719. doi: 10.1038/s41598-017-17739-7 (PMC5735156; doi:10.1038/s41598-017-17739-7)
Supplement: Supplementary file 1 — Supporting Information [file 41598_2017_17739_MOESM1_ESM.pdf]

Supplemental material for the manuscript

"Scattering of Ultra-relativistic Electrons in the Van Allen Radiation Belts Accounting for Hot Plasma Effects"

Xing Cao[1,2], Yuri Y. Shprits[2,3,4], Binbin Ni[1] and Irina S. Zhelavskaya[2,3]

[1] Department of Space Physics, School of Electronic Information, Wuhan University, Wuhan, China

[2] Helmholtz Centre Potsdam, GFZ German Research Centre for Geosciences, Potsdam, Germany

[3] Institute of Physics and Astronomy, University of Potsdam, Potsdam, Germany

[4] Department of Earth, Planetary, and Space Sciences, University of California, Los Angeles, California, USA

Supplementary Fig. 1 shows minimum resonant energies (MRE) as a function of L-shell and electron density for different abundances of hot protons corresponding to H^+ and He^+ band EMIC waves. All other parameters (ion concentration ratio, hot H^+ parallel temperature T_{hp} and hot H^+ temperature anisotropy) are identical to those used to obtain Figure 1. Although electron MRE for interactions with He^+ band EMIC waves decrease slightly with increasing hot proton density, for most typical values of electron density, resonant energies exceed 2 MeV. When the electron density is unusually high (above 1 standard deviation), MRE could be ~ 1.5 MeV.

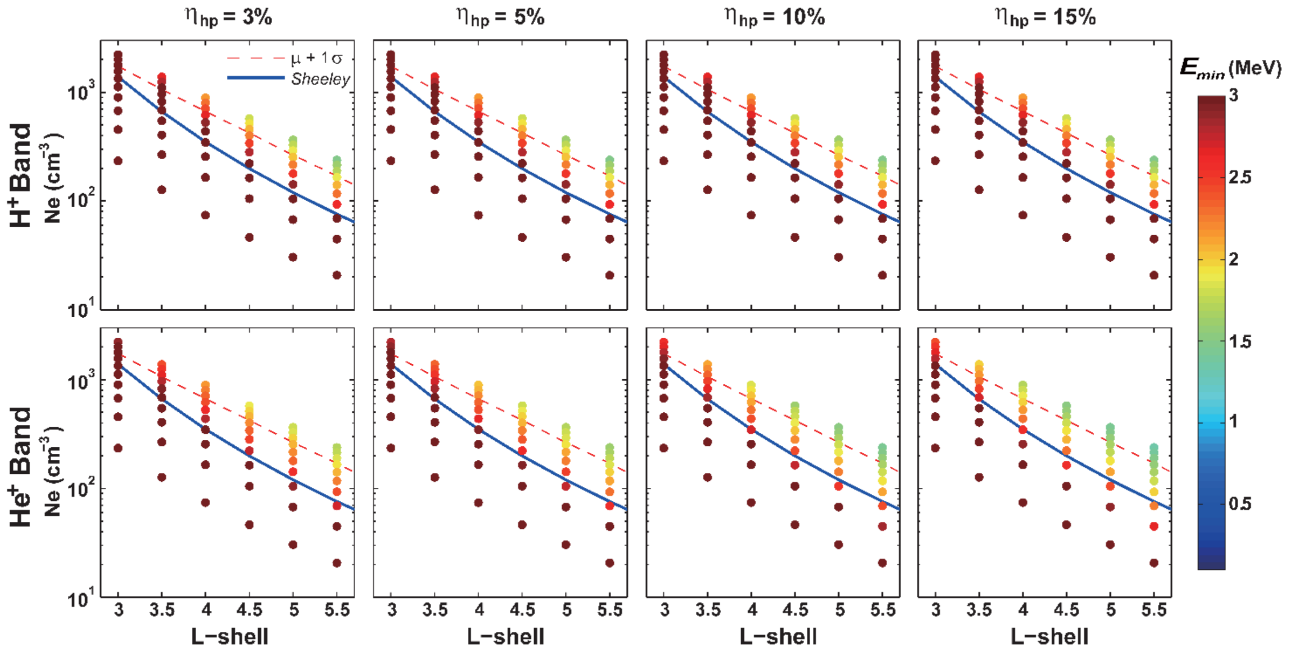

Supplementary Fig. 1. Sensitivity of electron MRE to hot H^+ abundance. Minimum resonant energies as a function of L-shell and electron density for different abundances of hot protons corresponding to H^+ and He^+ band EMIC waves.

Supplementary Fig. 2 shows minimum resonant energies (MRE) as a function of L-shell and electron density for different parallel temperature of hot protons corresponding to H^+ and He^+ band EMIC waves. All other parameters (ion concentration ratio, hot H^+ abundance η_{hp} and hot H^+ temperature anisotropy) are identical to those used to obtain Figure 1. It is shown that electron MRE for interactions with both H^+ and He^+ are insensitive to the variation of parallel temperature of hot protons.

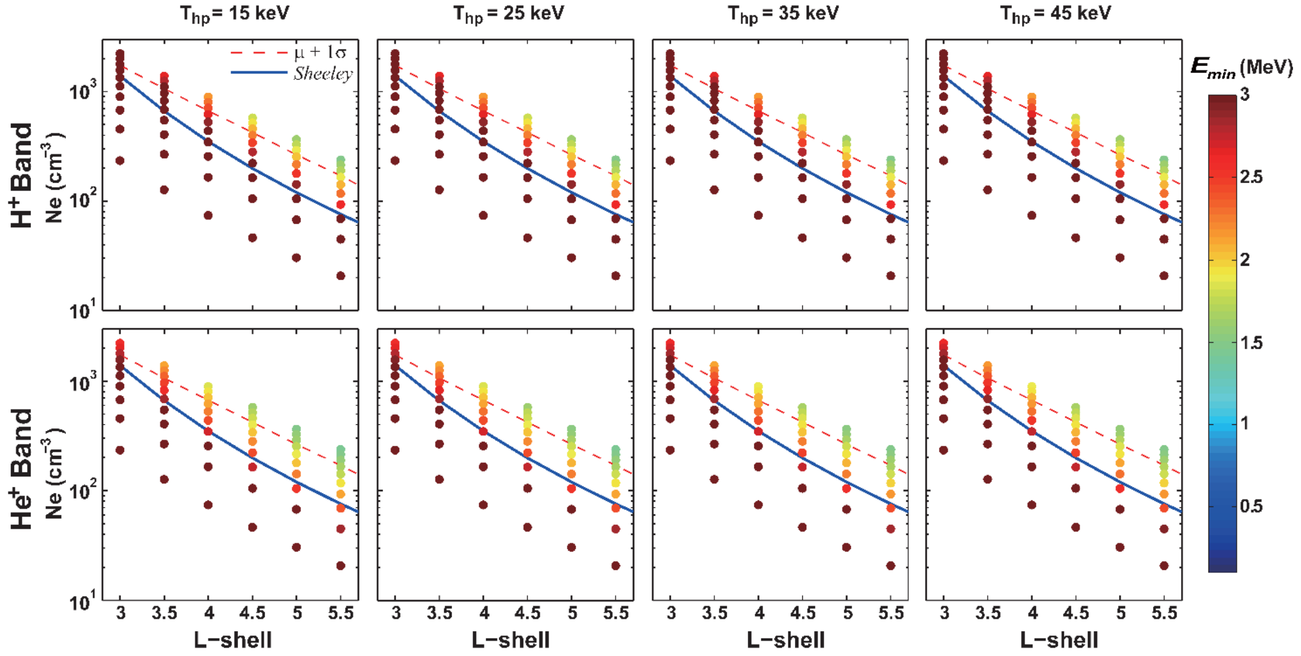

Supplementary Fig. 2. Sensitivity of electron MRE to the parallel temperature of hot protons. Same as in Supplementary Fig. 1, except for different parallel temperature of hot protons.

Supplementary Notes.

This study shows that electron MRE decrease with increasing radial distance and reaches the minimum at the upper limit of electron densities. Figure 2 illustrates that increase of temperature anisotropy of hot ring current protons lowers MRE for both H^+ and He^+ bands. There is a significant dependence of MRE on temperature anisotropy A_{hp} for H^+ band, while this dependence is much less pronounced for He^+ band. Figure 3 illustrates that an increase of O^+ ion abundance leads to a significant increase of MRE for both H^+ and He^+ band EMIC waves. Specifically, increasing the abundance of He^+ ions tends to slightly decrease MRE for He^+ band EMIC waves but increases MRE for H^+ band EMIC waves. Supplementary Fig. 1 and 2 illustrate that electron MRE is not very sensitive to the variation of hot proton abundance and parallel temperature. We demonstrate that, for all reasonable combinations of parameters, EMIC waves mainly contribute to the loss of ultra-relativistic electrons.

Previous observations also suggested that EMIC waves should be responsible for the precipitation loss of relativistic electrons. A recent study[4] provided observational support of MeV electron loss due to interactions with EMIC waves. It was also shown that EMIC waves could cause the loss of > 0.8 MeV electrons into the atmosphere[2]. It has been suggested that EMIC waves could even cause precipitation loss of electrons at energies down to 400-500 keV[3-5]. However, for many observational methods, finding

exact MRE, or exact energy of precipitating electrons may be a challenging task. It is also often difficult to separate precipitation simultaneously caused by different types of waves. As shown in this study, EMIC waves can resonate with MeV electrons when the electron density is much higher than the typical value and the hot proton anisotropy is very large. We also suggest that such precipitation, which should occur relatively rarely, is caused by scattering loss of very low equatorial pitch angle electrons and does not significantly influence the electron population. Other scattering mechanisms of radiation belt electrons at higher equatorial pitch angles, such as pitch angle scattering by magnetospheric chorus and / or hiss waves[6,7] or bounce resonance by EMIC waves[8,9], should be present simultaneously to cause significant loss of relativistic electron population.

Propagation effects of EMIC waves are not taken into account in this study. EMIC waves can propagate to higher latitudes along the geomagnetic field line after their generation near the equatorial region. At high latitude regions, EMIC wave spectra could be very close to or even across the gyro-frequencies of heavy ions owing to the increasing field intensities along magnetic field line; therefore, corresponding electron minimum resonant energies in particular can be lower than calculated results assuming growth rate $\gamma = 0$. However, once waves propagate into the region with $\gamma < 0$, they will be efficiently damped. It has been pointed out that EMIC wave energy will be absorbed efficiently at high latitudes via cyclotron damping near ion gyro-frequencies or bi-ion resonance frequencies[10-13].

Nonlinear effects play a significant role in the generation and saturation of

waves[14-16]. Rising or falling tones caused by the nonlinear wave growth are often observed in wave spectrograms. Nonlinear growth can change the wave frequency, and the corresponding wave growth can be even found in the region where linear growth rate $\gamma < 0$. However, the averaged spectral distribution will not be significantly altered, and we suggest that, $\gamma > 0$ is possibly a realistic assumption for modeling wave spectral properties for typical or frequent conditions. Understanding the potential role of EMIC wave nonlinear growth in the quantifications of electron MRE will be left to a future study.

The modification of EMIC wave dispersion relation caused by thermal effects not only changes the minimum resonant energy but also influences the evaluations of diffusion coefficients. We show in this study that inclusion of thermal effects can improve our current understanding of the energy range of resonant electrons. To evaluate more accurately the effect of hot plasma on loss, bounce-averaged scattering rates should be evaluated using hot plasma dispersion relation. A recent study[17] calculated the local diffusion coefficients of radiation belt electrons by EMIC waves using the kinetic linear dispersion relation. Nevertheless, considering the magnetic mirror geometry of the Earth's dipole-like magnetic field, bounce-averaged diffusion coefficients, which are obtained by averaging local diffusion coefficients over the particle bounce trajectory, need to be carried out to better understand the scattering loss of electrons by EMIC waves. Meanwhile, nonlinear resonant scattering of electrons could also be involved in the resonant interactions between EMIC waves and radiation belt electrons when the wave amplitude is strong enough[18,19]. It will be a subject of

future research to incorporate the thermal effects into calculations of diffusion coefficients and estimate the effects associated with the nonlinear scattering.

Supplementary references

1. Blum, L. W. *et al*. Observations of coincident EMIC wave activity and duskside energetic electron precipitation on 18–19 January 2013. *Geophys. Res. Lett.* **42,** 5727–5735 (2015).
2. Miyoshi, Y. *et al.* Precipitation of radiation belt electrons by EMIC waves, observed from ground and space. *Geophys. Res. Lett.* **35**, L23101 (2008).
3. Millan, R. M.*,* Lin*, R. P.,* Smith*,* D. M., Lorentzen*, K. R. &* McCarthy, M. P. X-ray observations of MeV electron precipitation with a balloon-borne germanium spectrometer. *Geophys. Res. Lett.* **29**(24), 2194 (2002).
4. Clilverd, M. A. *et al.* Electron precipitation from EMIC waves: A case study from 31 May 2013. *J. Geophys. Res. Space Physics,* **120**, 3618–3631 (2015).
5. Ni, B. *et al*. Dynamic responses of the Earth's radiation belts during periods of solar wind dynamic pressure pulse based on normalized superposed epoch analysis. *J. Geophys. Res. Space Physics.* **121**, 8523-8536 (2016).
6. Li, W., Shprits, Y. Y. & Thorne, R. M. Dynamic evolution of energetic outer zone electrons due to wave-particle interactions during storms. *J. Geophys. Res.* **112,** A10220 (2007).
7. Shprits, Y. Y., Chen, L. & Thorne, R. M. Simulations of pitch angle scattering of relativistic electrons with MLT-dependent diffusion coefficients*. J. Geophys. Res.*

114, A03219 (2009).

8. Shprits, Y. Y. Potential waves for pitch angle scattering of near-equatorially mirroring energetic electrons due to the violation of the second adiabatic invariant. *Geophys. Res. Lett.* **36**, L12106 (2009).
9. Cao, X. *et al*. Bounce resonance scattering of radiation belt electrons by H^+ band EMIC waves. *J. Geophys. Res. Space Physics.* **122**, 1702-1713 (2017).
10. Thorne, R. M. & Horne, R. B. Cyclotron absorption of ion-cyclotron waves at the bi-ion frequency. *Geophys. Res. Lett.* **20**(4), 317–320 (1993).
11. Thorne, R. M. & Horne. R. B. Modulation of electromagnetic ion cyclotron instability due to interaction with ring current O^+ during magnetic storms. *J. Geophys. Res.* **102**(A7), 14155–14163 (1997).
12. Horne, R. B. *&* Thorne, R. M. Wave heating of He^+ by electromagnetic ion cyclotron waves in the magnetosphere: Heating near the H^+-He^+ bi-ion resonance frequency. *J. Geophys. Res.* **102**(A6), 11457–11471 (1997).
13. Hu, Y., Denton, R. E. & Johnson, J. R. Two-dimensional hybrid code simulation of electromagnetic ion cyclotron waves of multi-ion plasmas in a dipole magnetic field. *J. Geophys. Res.* **115**, A09218 (2010).
14. Omura, Y. *et al*. Theory and observation of electromagnetic ion cyclotron triggered emissions in the magnetosphere. *J. Geophys. Res.* **115**, *A07234 (2010).*
15. Bortnik, J., Omidi, N., Chen, L., Thorne*, R. M. &* Horne, R. B. Saturation characteristics of electromagnetic ion cyclotron waves. *J. Geophys. Res.* **116***,* A09219 (2011).

16. Nakamura, S., Omura, Y. & Angelopoulos, V. A statistical study of EMIC rising and falling tone emissions observed by THEMIS. *J. Geophys. Res. Space Physics*. **121**, 8374–8391 (2016).

17. Chen, L., Thorne, R. M., Shprits, Y. & Ni, B. An improved dispersion relation for parallel propagating electromagnetic waves in warm plasmas: Application to electron scattering. *J. Geophys. Res. Space Physics.* **118**, 2185–2195 (2013).

18. Albert, J. M., & Bortnik, J. Nonlinear interaction of radiation belt electrons with electromagnetic ion cyclotron waves. *Geophys. Res. Lett.* **36***,* L12110 (2009).

19. Omura, Y. & Zhao, Q. Relativistic electron microbursts due to nonlinear pitch angle scattering by EMIC triggered emissions. *J. Geophys. Res. Space Physics.* **118**, 5008–5020 (2013).
